# Topological Octet-Rule Implementation for *Deltahedral* Boron Hydrides and Related Zintl Clusters of the Main-Group Elements: Flexible Octet-Rule Fulfillment by Mixed 2- and 3-Center Fractional Bonding Scenarios

**DOI:** 10.1021/acs.inorgchem.4c01390

**Published:** 2024-07-30

**Authors:** Frank R. Wagner, Yuri Grin

**Affiliations:** † Max Planck Institute for Chemical Physics of Solids, Chemical Metals Science, Nöthnitzer Straße 40, 01187 Dresden, Germany

## Abstract

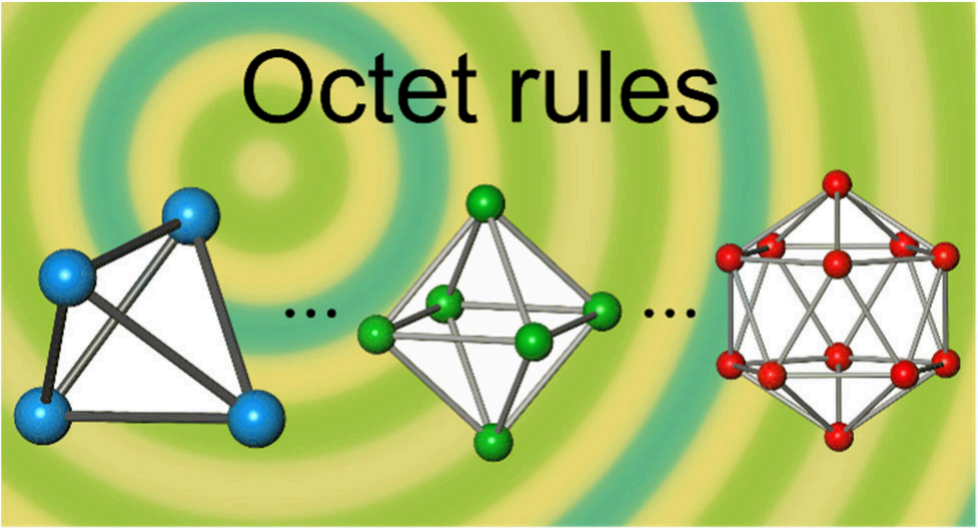

In the large class of main-group Zintl phases, the octet rule plays a key role for the polyanions following the pseudoatom concept and the 8–*N* rule, such that a unique correspondence between atomic partial structure and its electron count results. In the conceptual framework of the Wade’s type of clusters the relations to the octet rule are less obvious, and its structural implications are not clear. For this purpose, a topological implementation of the octet rule (TORI) within a delocalized bonding scenario is introduced. It is based on the average topology of the deltahedral cluster skeletons and the octet rule applied to delocalized fractional 2- and 3-center bond distributions. For a given skeletal electron pair count SEP, TORI yields values (*t*^toc^, *y*^toc^) similar to the *styx* approach. Two hierarchically different types of octet-rule fulfillment are identified, the cluster-wise and the local one. The local octet-rule fulfillment always implies the cluster-wise one, while the converse is not true. Deltahedral clusters with different skeletal shapes but the same Wade’s SEP count can be distinguished with respect to different octet-rule fulfillment. The TORI approach opens a perspective to compare Zintl phases containing Wade-type clusters with those containing 8–*N* type of partial structures on the basis of octet-rule implications. The main difference to the 8–*N* type of partial structures identified is the more flexible way of octet-rule fulfillment in the Wade’s type clusters, which does not prevent them from realizing the same cluster topology with different SEP counts. The TORI approach works with delocalized fractional bonds and is consistent with the concept of PSEPT; it just adds an additional facet.

## Abstract 2

The topological octet-rule implementation TORI closes the conceptual gap between main-group Zintl clusters following the 8−*N* and the Wade’s type of electron counting rules. It reveals two hierarchically different types of octet-rule fulfillment for deltahedral Wade’s type of clusters, and a more flexible way of octet-rule fulfillment not observed for 8−*N* type of partial structures.

## 1 Introduction

A large family of main-group element compounds can be conceptually understood from chemical bonding models that assume complete charge transfer from more electropositive components to electronegative ones (semimetals or nonmetals). The resulting anions form partial structures which can be explained by means of the electrons taken up from the cations. In one large group of these compounds the bonding connectivity (topology) in the polyanions follows the classical 8–*N* rule, which is a result of the Lewis octet rule.^1^ These represent Zintl phases in the most traditional sense of chemical bonding theory. In the Zintl–Klemm approach,^2^ the pseudoatom concept is used, i.e., the octet rule derived 8–*N* rule is applied to each pseudoatomic anionic species separately. This way the total number of electrons provided by the cations (assumption of complete charge transfer) is interpreted to yield a combination of pseudoelement anions, which in sum completely use these electrons to build up an electron-precise partial structure. An important feature of this framework, is that only 2-center 2-electron bonds are considered, and thus every anionic pseudoelement species features an integral number of 2-electron bonds and lone pairs which sum up to a value of 4. In a polar covalent framework, this notion is challenged, and coordination numbers larger than 4 are observed, e.g., in certain silicates where *CN*(Si) = 6, or e.g., CaF_2_ and TiNiSi type 8-valence electron main-group compounds with polar-covalent coordination numbers of 8 to 10. In these latter cases, the fractional covalent character of each bond, i.e., its fractional covalent bond population being smaller than 2 electrons, was shown to still yield local octet-rule fulfillment even for this type of bonding scenario in the framework of a polarity-extended 8–*N*^eff^ rule.^3^

Another group of main-group compounds obeys the Wade’s rules and modifications of it. Noteworthy, while the bonding model for the first group of compounds was already known in the time of Zintl (1898 - 1941), the basic ideas about the second group of compounds came up in the 1940s, and were further developed until the 1980s. For example, the cluster anion [Pb_9_]^4–^ with unknown constitution was identified as a component in a noncrystallizable Na_4_[Pb_9_] “polyanionic salt” solution in liquid ammonia by Zintl himself in 1931.^4^ The existence and established structures of the molecular boron hydrides, nonclassical carbo-cations, but also crystalline elemental boron, and metal borides played an enormous role as challenges for an appropriate bonding model.

In these main-group element clusters, even in the absence of polar bonding, the number of covalently bonded neighbors of the atomic species exceeds the number of electrons available for 2-center 2-electron bonding. For this reason, they were often characterized as electron-deficient molecules. Covalent coordination numbers typically larger than 4 is another characteristic feature of these compounds. Moreover, the spatial arrangements of those neighbors are not consistent with the 8–*N* type of bonding model.

The introduction of the 3-center 2-electron bond formalism^5,6^ has been the decisive development to explain bonding scenarios, where the number of electrons is too short to form 2-center 2-electron bonds between all covalently bonded neighbors. With its description of the cluster bonding as a sum of local 2- and 3-center 2-electron bonds, the conceptual framework built up by the solutions of hydrogen, orbital and charge balance equations and encoded into the Lipscomb’s *styx* code^7^ was shown to have predictive power for structures of known and compositions of novel boron hydride compounds.^8^ In this code, parameter *s* denotes the number of skeletal 3-center 2-electron B–H–B bonds, *t* the number of skeletal 3-center 2-electron B–B–B bonds, *y* the number of skeletal 2-center 2-electron B–B bonds, and *x* the number of extra (in addition to the always present exohedral bond per skeletal atom) 2-center 2-electron B–H bonds. The condensation of the *styx* code to just the number of skeletal electron pairs (SEP)
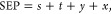
1and the observation that SEP already correlates with the geometric structure for a given number of skeletal atoms,^9^ may be considered as an information-reduction step^10^ avoiding local bonding complexities. With this simplification, advancement toward systematic understanding of composition and atomic arrangement was clearly evidenced, namely relating the genealogy of *closo*- *nido*-, and *arachno*-skeletons to their SEP numbers.^9^ This step also marked a change of methodology and reasoning, from a (semi-) localized orbital derived extension of the Lewis-type picture keeping with the octet rule to the pure molecular orbital (MO) picture based solely on delocalized canonical molecular orbitals and their energies. The Wade’s rules are the result of the electron count for skeletal bond saturation,^9,11^ which can be obtained from population analysis of canonical MO wave functions. Since the skeletal bond saturation criterion is very easy to analyze in actual MO calculations, it represents the standard numerical approach to analyze cluster bonding in molecules and solids nowadays. The MO theory behind the Wade’s rules, i.e. the decisive importance of number of occupied skeletal bonding orbitals SEP, has been later explained on the basis of an approximate spherical MO approach called the spherical tensor surface harmonic (TSH) model of Stone.^12^ On the basis of MO approaches Mingos has outlined a scheme called the Polyhedral Skeletal Electron Pair approach PSEPT, which attempts to unify polyhedral molecules featuring localized 2-center 2-electron bonds with the Wade’s type of clusters with delocalized bonding.^13^ For this purpose, the polyhedral electron count (PEC) summarizing the skeletal (SEP) and exohedral electron pairs (bonds or lone pairs) was introduced as the decisive electronic cluster characteristic. A useful summary of all these and many more achievements can be found in ref (14). The type of compounds successfully analyzed with the Wade-Mingos concept ranges from borane, carborane, and carbocation compounds to metallaboranes and organometallic compounds, where the transition metal species are expected to follow the isolobal concept.^15^ In the present context, it is important to realize that none of these MO models explicitly relates its results for Wade type clusters to the octet rule.

**Figure 1:**
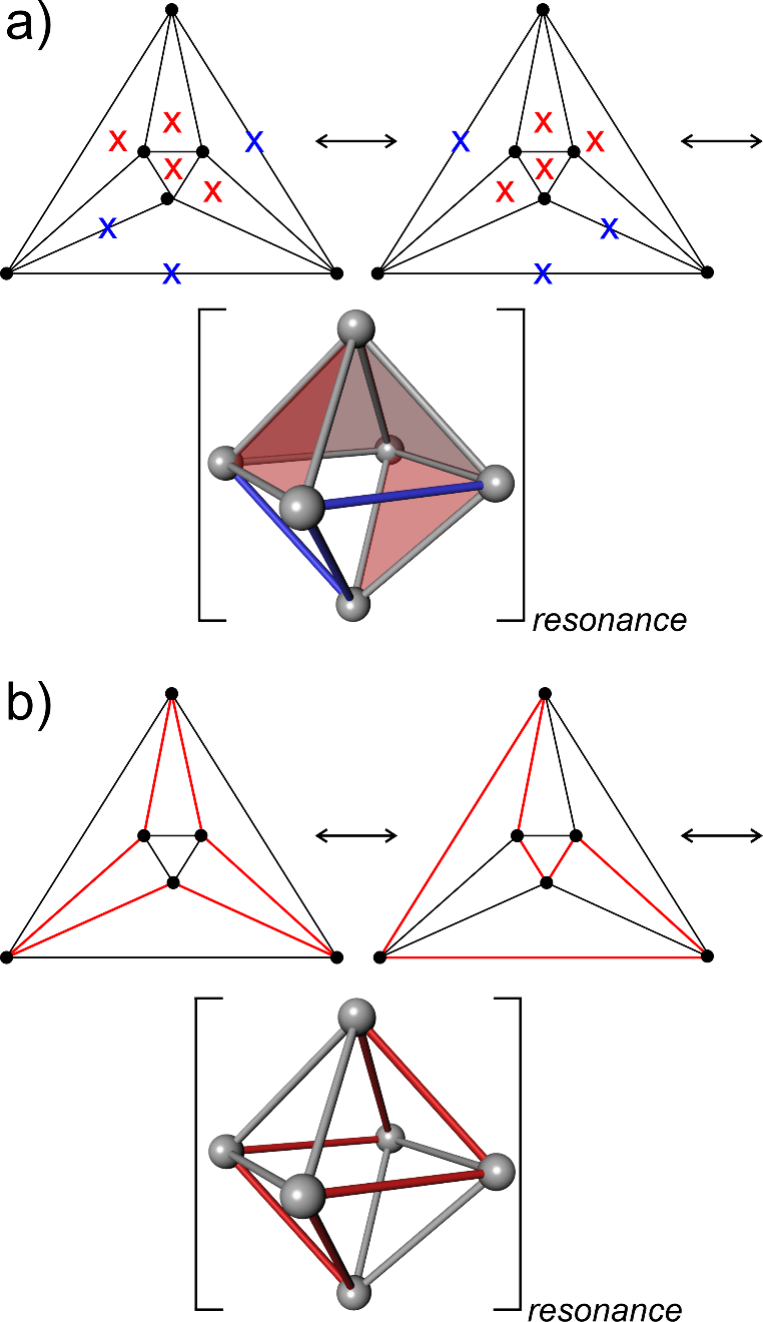
Schlegel diagrams and 3d representation of the *closo* octahedral cluster skeleton like in B_6_H_6_^2–^ with *styx* code (0, 4, 3, 0): (a) resonance between different local octet realizations is shown by crosses “*x*” on allocated faces (*t* = 4, red) and edges (*y* = 3, blue); and (b) resonance of Hamiltonian circuits (red lines).

From the viewpoint of the Zintl phases, it is interesting to note that the active use of the 8–*N* rule always reminds one of the important conceptual role of the octet rule. At first glance, this looks different for Zintl phases consisting of charged bare main-group clusters isoelectronic with Wade’s type clusters and with an identical or related skeletal structure. The Wade’s SEP number corresponds to the global cluster quantity dominantly characterizing its main electronic properties in a chemical sense. The octet rule is locally related to each skeletal specie’s bonding situation within the cluster. The overall optimization of local bonding situations within the restrictions posed by the whole cluster unit finally yields the cluster’s SEP number. However, supplementing Lipscomb’s topological bond data on *closo* boron hydride skeletons,^7^ Wade^16^ has explicitly investigated and verified, that the *styx* code numbers of 2-center 2-electron bonds and 3-center 2-electron bonds can be realized in the known deltahedral *closo* skeletons of B_*n*_H_*n*_^*2*–^ (*n* = 5 to 12) in such a way that they fulfill the local octet rule.^17^ In this sense, for all main-group clusters with the prototype *closo* cluster topology and a SEP^closo^ count, the local octet-rule fulfillment has been already verified in the past (Figure 1a, example for octahedral cluster).

Furthermore, in an approach complementary to the *styx* one, the graph theoretical interpretation of the bonding topology in deltahedral boranes based on Wade’s orbital model for these clusters^9b^ resulted in King’s graph theoretical bonding model.^18^ Here, the *n* bonding tangential orbitals form *n* 2-center 2-electron bonds along the deltahedral edges, which are arranged in closed loops and meet each vertex exactly once (Hamiltonian circuits in the deltahedral graph, Figure 1b). These bond circuits are considered to be equally distributed over all deltahedral edges by resonance. It is this resonance feature that King’s model has in common with the *styx* approach. In the complete model, one cluster-core bond representing an *n*-center 2-electron bond is added per *closo*-cluster. In sum, each skeletal atom in a *closo*-borane like cluster is invoked into one exo-bond (or lone pair), two 2-center 2-electron bonds, and one *n*-center 2-electron bond. Thus, this model is consistent with the local fulfillment of the octet rule as well.

It is incomprehensible that the octet rule for atomic arrangements of main-group elements should sharply disappear on the way from the 8–*N* type polyanions to the clusters of the Wade-type bonding schemes. Thus, the goal of the present study is to examine what has happened to the octet-rule concept in the Wade’s clusters. For this purpose, a bonding approach for the typically delocalized cluster bonding in deltahedral clusters is developed, which is based on the cluster topology and the octet rule, without the recourse to orbitals.

## 2 Results and Discussion

### 2.1 Topological Features of the *Closo*-clusters’ Skeletal Structures

**Figure 2:**
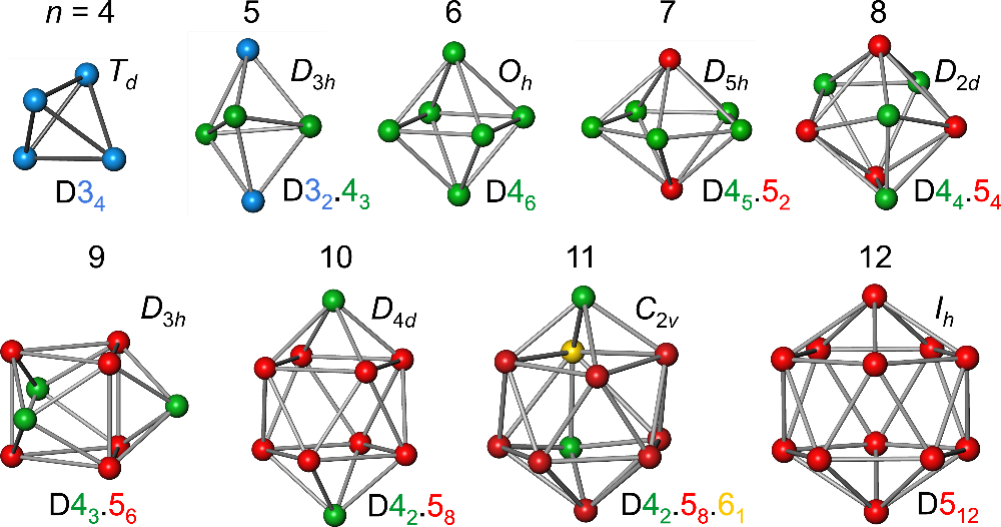
Skeletal structure representations, symmetries and vertex compositions D3_*q*_.4_*r*_.5_*s*_.6_*u*_ of B_4_H_4_ and B_*n*_H_*n*_^2–^*closo*-clusters (*n* = 5 to 12); colors of vertex compositions are consistent with vertex colors in graphics.

The *n*-vertex skeletons of B_4_H_4_ and the *closo*-borane clusters B_*n*_H_*n*_^2–^ (*n* = 5 to 12) (Figure 2) and related compounds display a deltahedral topology. In this sense, they are all denoted as “deltahedra” in the present context. This means that the skeletal connections of these convex polyhedra exclusively form triangular faces, which are not necessarily regular (equilateral). The highly symmetrical deltahedra with regular triangular faces and congruent vertices are the tetrahedron (*n* = 4), the octahedron (*n* = 6), and the icosahedron (*n* = 12), which belong to the platonic solids with point group symmetries *T*_*d*_, *O*_*h*_ and *I*_*h*_, respectively.

With *n*_V_ (= *n*), *n*_E_, and *n*_F_ representing the numbers of polyhedral vertices, edges, and faces, respectively, the polyhedral Euler equation takes the form
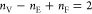
2From the deltahedral topology the ratio of the numbers of edges and faces of a deltahedron is given according to
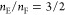
3

By additionally considering, that for an *n*-vertex deltahedron the numbers of edges and faces are given already by the polyhedral size *n* (= *n*_V_, eq 2), one obtains
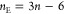
4
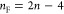
5The degree *vd* of a vertex enumerates its number of connections (edges meeting at that vertex).^19^ The vertex degrees of the *n* = 4, 6, 12 *closo* deltahedra are uniform, i.e., they are identical for each vertex of the corresponding deltahedron, and display values of 3, 4 and 5, respectively. This way, the *closo* 4-, 6-, and 12-vertex deltahedra can be characterized by their vertex-degree compositions D3_4_, D4_6_, and D5_12_, where the subscript enumerates the number of vertices of given degree *vd* (Figure 2). In general, the *closo* deltahedral clusters with their *n*-vertex skeletons can be characterized by their specific vertex composition D3_*q*_.4_*r*_.5_*s*_.6_*u*_, where *n* = *q* + *r* + *s* + *u*. From the number of edges of an *n*-vertex deltahedron (eq 4), the average vertex degree  can be computed for each deltahedron.
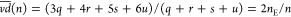
6Substituting *n*_E_ by eq 4 it can be seen, that  increases monotonically with *n*:  = 6 (*n*–2)/*n* (Table 1).

**Table 1:** Solutions of Deltahedral TORI Equation for Different Skeletal Sizes *n* and Electron Counts 2SEP(*n*)^a^

*n*	2SEP = 2(*n* – 1)	2SEP = 2*n hypercloso*	2SEP = 2(*n* + 1) *closo*	2SEP = 2(*n* + 2) *nido*	2SEP = 2(*n* + 3) *arachno*	2SEP = 2(*n* + 4) *hypho*	2SEP = 2(*n* + 5) not named
4	./.	8, 0	4, 6	0, 12	./.	./.	./.
		1	0.4	0			
		(4, 0)	(2, 3)	(0, 6)			
5	./.	10, 0	6, 6	2, 12	./.	./.	./.
		1	0.5	0.143			
		(5, 0)	(3, 3)	(1, 6)			
6	./.	12, 0	8, 6	4, 12	0, 18	./.	./.
		1	0.571	0.25	0		
		(6, 0)	(4, 3)	(2, 6)	(0, 9)		
7	./.	14, 0	10, 6	6, 12	2, 18	./.	./.
		1	0.625	0.333	0.1		
		(7, 0)	(5, 3)	(3, 6)	(1, 9)		
8	./.	16, 0	12, 6	8, 12	4, 18	0, 24	./.
		1	0.667	0.4	0.182	0	
		(8, 0)	(6, 3)	(4, 6)	(2, 9)	(0, 12)	
9	./.	18, 0	14, 6	10, 12	6, 18	2, 24	./.
		1	0.7	0.455	0.25	0.0769	
		(9, 0)	(7, 3)	(5, 6)	(3, 9)	(1, 12)	
10	./.	20, 0	16, 6	12, 12	8, 18	4, 24	0, 30
		1	0.727	0.5	0.308	0.143	0
		(10, 0)	(8, 3)	(6, 6)	(4, 9)	(2, 12)	(0, 15)
11	./.	22, 0	18, 6	14, 12	10, 18	6, 24	2, 30
		1	0.75	0.538	0.357	0.2	0.0625
		(11, 0)	(9, 3)	(7, 6)	(5, 9)	(3, 12)	(1, 15)
12	./.	24, 0	20, 6	16, 12	12, 18	8, 24	4, 30
		1	0.769	0.571	0.4	0.25	0.118
		(12, 0)	(10, 3)	(8, 6)	(6, 9)	(4, 12)	(2, 15)
*n*	./.	2*n*, 0	2(*n*–2), 6	*2(n*–4), 12	2(*n*–6), 18	2(*n*–8), 24	2(*n*–10), 30
		*n*/*n*	(*n*–2)/(*n*+1)	(*n*–4)/(*n*+2)	(*n*–6)/(*n*+3)	(*n*–8)/(*n*+4)	(*n*–10)/(*n*+5)
		(*n*, 0)	(*n*–2, 3)	(*n*–4, 6)	(*n*–6, 9)	(*n*–8, 12)	(*n*–10, 15)

a For each solution (one cell), the values *t*^toc^, *y*^toc^ (1st line), *γ* (2nd line), and *styx* type of values *t* and *y* (3rd line) are given; chemically valid solutions obey 0 ≤ *γ*(*n*) ≤ 1, non-valid solutions are indicated by “./.”.

It has been shown^20^ that for *n* = 6–10, and 12 there exists only one topologically distinct deltahedron of each size *n*, with (*q*, *u*) = 0 and (*r*, *s*) ≥ 0. In sum, for all *n*-vertex deltahedra (*n* = 6 to 10) realized by the boron hydride *closo n*-skeletons, the specific vertex composition D4_*r*_.5_*s*_ uniquely specifies this deltahedral topology. For a given deltahedral size *n* various other deltahedra with different topologies typically exist; they all have a vertex composition different from the boron hydride *closo*-skeleton one.

The observation, that existing homoatomic *closo*-boron hydride clusters prefer vertex compositions as uniform as possible points to an energetic advantage of the associated bonding situation. Their deltahedral shapes are considered as the prototypes for *closo n*-vertex clusters. The realization of nonligated (bare) main-group element clusters [*E*_*n*_]^*q*^ in polar intermetallics with a Wade-type *closo* electron count (SEP = *n* + 1), and a total polyhedral electron count (including the exo-skeletal lone pairs) of PEC = 4*n* + 2 ve,^13^ was sometimes found to exhibit structural deviations with respect to the boron hydrides. Although being isoelectronic and isolobal, the basic cluster building blocks [:B–H] and [:*E*–lp] (lp = lone pair) are found to not always behave in a completely identical fashion.^21^ In these cases, the deltahedral cluster *n*-skeletons display a vertex composition different from the boron hydride ones with the same number of vertices. Nevertheless, the average vertex degrees are identical with the prototype ones because of eqs 4 and 6 (second identity).

### 2.2 3-Center Ratios Γ(*n*) for *closo styx* Values

For boron hydrides and related systems, the chemical bonding scenario has been characterized as a mixture of 2-center and 3-center bonding as encoded in the *styx* code.^6,7^ For the *closo*-boranes B_*n*_H_*n*_^*c*–^ (*n* = 5 to 12) with *c* = 2 and *s* = *x* = 0, the orbital (eq 7) and charge balance (eq 8) equations (OCBE)^6b^
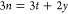
7
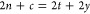
8directly yield a general relation between the cluster size *n* (*n* = 5 to 12) and the numbers *y* and *t* of two- and three-center bonds in the cluster with *closo* electron count,^7^ respectively:
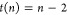
9
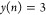
10Here, the sum *t* + *y* = SEP, is the Wade’s number of skeletal electron pairs. Eqs 9 and 10 describe a systematic behavior of the 2- and 3-center characters of the bonding with increasing cluster size *n* (Table 1). The noncharged (*c* = 0) tetrahedral B_4_H_4_ type of clusters represent an exceptional case (*hypercloso* type of skeletal electron count), where the OCBE correctly yield *t* = 4, *y* = 0.

The *styx* approach has been classified as a (semi)topological one,^7^ because it works with a topological allocation of four types (*s*, *t*, *y*, *x*) of 2-electron bonds between the skeletal atoms. It is important to realize that the resonance smearing of these bond allocations to account for symmetrically equivalent realizations finally destroys this topological bond picture.

Since a deltahedron with *n* vertices displays (3*n*–6) edges (eq 4), and (2*n*–4) triangular faces (eq 5) on the surface, for each additional: B–H vertex supplying 2 additional skeletal electrons three additional edges are created, which systematically increases electron deficiency with respect to 2-center 2-electron bonding along the edges. Within the *styx* concept, this is compensated by additional 3-center 2-electron bonding, which is supported by concomitant creation of 2 new triangular faces. From a delocalized point of view, the ratio of the number of 3-center 2-electron bonds and available triangular faces of the deltahedra (*n* – 2)/(2*n* – 4) = 0.5 yields a constant occupation of 50% of all deltahedral triangles by 3-center 2-electron bonds for *n* = 5 to 12. In contrast, only three of the 3*n* – 6 edges are occupied by 2c-2e bonds, which results in a systematically decreasing fractional 2c-character of 3/(3*n* – 6) = 1/(*n* – 2) per edge associated with increasing number of vertices along *n* = 5 to 12.

The conceptual 3-center ratio Γ(*n*) is now defined according to eq 11.
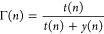
11It clearly shows the trend of increasing portion of 3-center bonding with increasing cluster size *n*. This means, for *closo*-boranes (*s* = 0, *x* = 0) with SEP(*n*) = *t*(*n*) + *y*(*n*) and related compounds with *styx* = (0, *t*, *y*, 0), knowledge of Γ and SEP is sufficient to calculate *t* and *y* ():
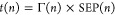
12
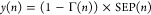
13Suitable local arrangements for these numbers of 2- and 3-center 2-electron bonds may still follow the octet rule and represent an extension of the Lewis concept. An important point in this respect is that the actual arrangement of such bonds depends on the specific polyhedral topology, i.e., the exact distribution and interconnection of vertices with their specific vertex degrees (the number of edges meeting at a vertex). Up to now, no explicit mathematical expression has been given that would immediately give an answer as to whether a certain number of 2- and 3-center 2-electron bonds can be distributed on the surface of a specific polyhedron in an acceptable way consistent with the octet rule. This is usually explicitly checked by testing for each case. For the *closo*-boron hydride skeletons (*n* = 5 to 12), initial investigations by Lipscomb^7^ were complemented by Wade,^16^ who proved the fulfillment of the local octet rule for *closo* electron count by showing acceptable arrangements of three 2-center and (*n* – 2) 3-center 2-electron bonds in these skeletons. Considering that the final cluster bonding picture corresponds to a resonance of all relevant bond allocations such that finally the 2-electron character for each edge or face bonding is lost, the question arises about the importance of the localized bond allocations and the chemical guidelines for their construction^16^ within the delocalized-bonding framework in these clusters.

A deeper view on the OCBE reveals that they only separately specify, that besides the omnipresent exo-bond (or lone pair) all the remaining 3 orbitals of each skeletal main-group atom are used in 2- and 3-center skeletal bonding (orbital balance), and, that all electrons associated to skeletal bonding are used for this purpose in a way to yield in sum integral net populations of 2-electron bonds for each bonding-centricity type (charge balance). As a final result, this yields a closed valence shell around each skeletal atom, i.e., a rather general implementation of the octet rule. The usual way to interpret the OCBE by employment of integral bond entities, the 2- and 3-center 2-electron bonds, corresponds to an interpretation of the OCBE in a Lewis-type (the rule of two) sense (case A). A more general interpretation (case B) would be to allow also fractional skeletal bonds with nonintegral electron numbers that only finally sum up to integral numbers of 2-electron bonds of 2- and 3-center types. This corresponds more to the advanced analysis of these localized bond arrangements by Lipscomb, where fractional 3-center bonds have been finally proposed to reconcile his results from orbital localization procedures with the *styx* framework of localized 2- and 3-center 2-electron bond arrangements. It has been emphasized that such a replacement does not affect the effective 4-valency of the skeletal main-group species.^22^

The computability of *styx*-type *t*, *y* values from a given Γ value (eqs 12 and 13) opens the way to a more general interpretation of *t* and *y* values as a sum over fractional delocalized and not necessarily 2-electron localized bonds (case B mentioned above) because only the ratio Γ of total 2- and 3-center bonds is employed. As described below, this idea is the basis for computing (*t*^toc^, *y*^toc^) values in the framework of the topological octet rule implementation (TORI), which are then compared to the *styx* (*t*, *y*) ones.

### 2.3 TORI Approach

#### 2.3.1 TORI Equation

In this section, it will be shown that the characteristic overall edge and face bond-occupations for deltahedral cluster skeletons like the *closo*-borane ones with vertex compositions D3_*q*_.4_*r*_.5_*s*_.6_*u*_ can be derived from the deltahedron’s topology, the skeletal electron numbers 2SEP(*n*), and the octet rule within a delocalized bonding scenario, i.e., 2-electron bonds are not assumed. This ansatz is complementary to the localized bonding scenario of the *styx* approach, where the initial condition of boron using all its 4 atomic orbitals for exohedral and skeletal bonding implicitly induces the octet rule from sums of localized 2-electron bonds (OCBE eqs 7 and 8).^6b,7^

For the topological implementation of the octet rule for main-group element deltahedral clusters, an equation is derived, where variable 0 ≤ γ(*n*) ≤ 1 represents the mixing coefficient between two complementary scenarios, either (i) complete distribution of 2SEP(*n*) electron on all deltahedral faces, or (ii) complete distribution of 2SEP(*n*) electrons on all deltahedral edges. With the former distribution typically overshooting the octet rule, and the latter one under-fulfilling it, γ(*n*) is determined as the mixing coefficient between both situations that leads to a fulfillment of the octet rule, keeping in mind, that each skeletal atom already has 2 electrons assigned from the *exo*-bond.

Thus, imposing octet-rule fulfillment of the average skeletal vertex with average skeletal vertex degree  by adequately mixing its electronic edge and face (triangle) bond-occupations yields the topological octet rule implementation (TORI) given in eqs 14 and 15:
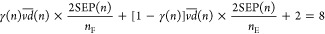
14with solution
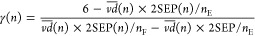
15 – average vertex degree of an *n*-vertex deltahedron (eq 6);

*n*_F_ – number of faces of an *n*-vertex deltahedron (eq 5);

*n*_E_ – number of edges of an *n*-vertex deltahedron (eq 4);

SEP(*n*) – number of skeletal electron pairs of Wade (eq 1);

γ(*n*) – overall mixing coefficient between skeletal edge allocation (γ(*n*) = 0) and skeletal face allocation (γ(*n*) = 1) of the 2SEP(*n*) skeletal electrons;

Chemically valid solutions for the mixing coefficient obey 0 ≤ γ(*n*) ≤ 1.

The corresponding overall values of skeletal face-allocated *t*^toc^ and edge-allocated *y*^toc^ electrons (summing up to 2SEP(*n*)) are obtained according to
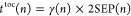
16
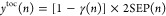
17Note that eqs 16 and 17 are besides a factor of 2, the same as in the OCBE framework (eqs 12 and 13). The factor of 2 is related to the fact, that electron pairs are not explicitly employed in the TORI framework, and it is just consistent to employ *t*^toc^, *y*^toc^ in terms of electrons and not electron-pairs.

#### 2.3.2 Solution Spectrum of the TORI Equation

The spectrum of chemically valid solutions (0 ≤ γ(*n*) ≤ 1) of the TORI equations for deltahedral skeletal topology (eqs 14–17), in short, the TORI deltahedral solution spectrum, is given in Table 1. It can be seen, that there is a clear lower boundary for all skeletal sizes *n* at 2SEP = 2*n*, below which the TORI equation cannot be fulfilled. This means for lower 2SEP numbers, the octet rule cannot be fulfilled within the mixed 2- and 3-center bonding approach at the deltahedral surface. Higher bonding centricity or introduction of interstitial atoms are then possible solutions for those situations. For example, the filled icosahedral skeleton of the Al_13_^1–^ cluster^23^ has a total polyhedral electron count of 40 ve: assuming 13 exo-electron pairs for a hypothetical 13-vertex deltahedron, a corresponding SEP = 7 would be obtained being much smaller than SEP^hypercloso^(13) = 13. It is conceptually explained with the jellium model.^24^.

Interestingly, the upper boundary systematically increases with the cluster size. The range of allowed solutions covers the conceptually known scenarios of the *hypercloso* (2SEP = 2*n*), *closo* (2SEP = 2(*n* + 1)), *nido* (2SEP = 2(*n* + 2)), *arachno* (2SEP = 2(*n* + 3)), and *hypho* (2SEP = 2(*n* + 4)) electron counts. The *hypho* count represents the final scenario only up to *n* = 9, for larger skeletons further scenarios, e.g. with 2SEP = 2(*n* + 5) are predicted.

The feature in focus of the present study, is the part of the solution spectrum (γ(*n*), *t*^toc^(*n*), *y*^toc^(*n*)) obtained for the deltahedral cluster skeletons with hypercloso, *closo*, and *nido* skeletal electron counts. It is identical with the solutions of the *styx* OCBE for these electron counts.^16^ In contrast to Wade’s results showing nonregular behavior for local octet-rule fulfillment in the deltahedral *hypercloso* and *nido* cases, the cluster-wise octet rule-fulfillment obtained from TORI is always satisfied.

A more detailed discussion of the TORI solution spectrum, especially the relation between the (*t*^toc^, *y*^toc^) solutions obtained for non-*closo* skeletal electron counts and their realization in nondeltahedral skeletons is beyond the scope of the present study.

#### 2.3.3 Two Types of Octet-Rule Fulfillment from TORI Approach

Although the local vertex bonding situations typically display fractional bonds (see examples below), the sum of them over the full cluster skeleton yields integral numbers *t*^toc^(*n*) and *y*^toc^(*n*) identical to 2*t*(*n*) and 2*y*(n) of the *styx* approach for skeletal *closo* clusters with *closo* electron count 2SEP^closo^ (Table 1):
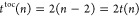
18
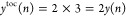
19Thus, the values *t*^toc^(*n*), *y*^toc^(*n*) obtained from eqs 16 and 17 exactly reproduce the well-known *t*(*n*), *y*(*n*) relations known from the *styx* approach (eqs 9 and 10) for *closo n*-vertex deltahedra (*n* = 5 to 12). This correspondence between the TORI type of bonding parameters and the *styx* type ones, i.e., γ(*n*) = Γ(*n*), such that *t*^toc^(*n*) = 2*t*(*n*), and *y*^toc^(*n*) = 2*y*(*n*), points to the interpretation of the generalization of type B mentioned above for the *styx* orbital and charge balance equations (eqs 7 and 8). Both approaches, while starting from complementary assumptions, are mutually consistent. Thus, the combined picture of both approaches has more facets than each separate one.

For the subsequent discussion of two types of octet-rule fulfillment, a cluster-wise and local one, more compact working equations are used. Rewriting eq 14 using eqs 16 and 17 yields the cluster-wise octet-rule fulfillment of the cluster-averaged vertex-configuration count  in terms of the cluster-averaged vertex degree , and (*t*^toc^, *y*^toc^) values (eq 20).
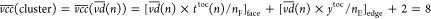
20While eqs 14 and 20 indicate that cluster-wise octet-rule fulfillment is always true in the TORI framework, local octet-rule fulfillment is not guaranteed.

In order to test local octet-rule fulfillment of the atomic species at vertex *v*_*i*_ a similar type of equation can be used, where the average vertex degree  is substituted by the vertex degree *vd*(v_*i*_) of the actual vertex v_*i*_ (eq 21).
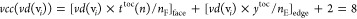
21

The key point in this equation is the electronic occupation of the local edges and faces connected to *v*_*i*_. The way of calculation of the face and edge occupations in eq 21 yields homogeneous occupations, because all connected faces and edges obtain the same electronic occupation of *t*^toc^/*n*_F_, and *y*^toc^/*n*_E_, respectively. However, homogeneous occupation is only required if all faces and edges are symmetrically equivalent. If they are symmetrically different, then the electronic face and edge occupations *focc*(*j*) and *eocc*(*j*), respectively, become free parameters, which can be adjusted to yield local octet-rule fulfillment:
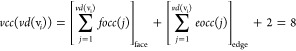
22

Since all *n* vertices have to fulfill such an equation simultaneously, the electronic face and edge occupations, *focc*(*i*, *j*) and *eocc*(*i*, *j*), respectively, must fulfill a sum rule:
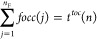
23
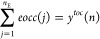
24

Local octet-rule fulfillment is indicated, when the value obtained for the local vertex-configuration count *vcc*(*vd*(v_*i*_)) is equal to 8 ve (eq 22). Even if this is not possible for all vertices, the cluster-wise octet-rule is always fulfilled according to eq 20, which can be rewritten as the arithmetic average of all local vertex-configuration counts (eq 25):
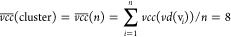
25

In general, because of the usage of the average vertex degree , eqs 14 and 15 include the topology of a deltahedral *closo n*-vertex cluster in an average way. An important consequence of this feature is, that the average vertex degrees used to calculate γ(*n*), not only describe the *n*-vertex deltahedra observed for the *closo* boron hydrides. Other *n*-vertex deltahedra with different vertex composition D3_*q*_.4_*r*_.5_*s*_.6_*u*_ (*n* = *q* + *r* + *s* + *u*) still have the same number of edges and thus the same  value because of eq 6. This is important, because not all bare Zintl-clusters display the same deltahedral topology as the boron hydrides. For an *n*-vertex *closo* cluster with the same deltahedral skeletal size *n* as the corresponding prototype boron hydride one (Figure 2), the same *t*^toc^, *y*^toc^ values are obtained. These values always fulfill the cluster-wise octet rule (eqs 20 and 25). In those cases with a skeletal topology identical to the prototypes boron hydrides, they need not be tested for local octet-rule fulfillment, since Wade has already verified this aspect.^16^ For alternative *n*-vertex deltahedra with the same SEP(*n*) the fulfillment of the local octet rule has to be checked explicitly for each vertex type. All of this will be illustrated by a few examples in the following.

#### 2.3.4 Examples

Table 2 summarizes the results for the cases exemplarily discussed below and a few other examples of Zintl-clusters. Note, in contrast to the formulas given above, the “ve” units are always written in the following for the sake of better understanding the calculations.

**Table 2:** TORI and *Styx* Analysis of Deltahedral Main-Group Clusters^a^

*n*	vertex comp.		SEP	*t*, *y* OCBE	Γ = γ	*t*^toc^, *y*^toc^ TORI	Octet rule fulfillment clust./loc.	examples
4	D3_4_	3	4	4, 0	1	8, 0	yes/yes	B_4_Cl_4_ ^25^
			6	0, 6	0	0, 12	yes/yes	*A*^(1)^_4_[Si_4_]^4–^ ^26^
5	D3_2_.4_3_	3.6	6*	3, 3	0.5	6, 6	yes/yes	[*E*^(14)^_5_]^2–^ ^27^
6	D4_6_	4	7*	4, 3	0.571	8, 6	yes/yes	CaB_6_ ^28^
7	D4_5_.5_2_	4.286	8*	5, 3	0.625	10, 6	yes/yes	—
8	D4_4_.5_4_	4.5	9*	6, 3	0.667	12, 6	yes/yes	—
	D3_4_.6_4_						yes/no	[Ge_8_]^2–^^29^
9	D4_3_.5_6_	4.667	10*	7, 3	0.7	14, 6	yes/yes	[Ge_9_]^2–^ ^20^
			9	9, 0	1	18, 0	yes/yes	B_9_Cl_9_^31^
			11	5, 6	0.455	10, 12	yes/yes	[Bi_9_]^5+^ ^27^
10	D4_2_.5_8_	4.8	11*	8, 3	0.727	16, 6	yes/yes	[Pb_10_]^2–^ ^32^
								[TlSn_9_]^3–^ ^27^
11	D4_2_.5_8_.6_1_	4.909	12*	9, 3	0.75	18, 6	yes/yes	—
12	D5_12_	5	13*	10, 3	0.769	20, 6	yes/yes	[Pb_12_]^2–^^33^

a SEP^closo^ count is marked by an asterik “*”; cases for which no examples have been found are indicted by “—”. *Styx* values (0, *t*, *y*, 0) are obtained from *closo* or other electron-pair counts SEP^closo^(*n*) ± 1 for deltahedral *n*-vertex clusters using orbital and charge-balance equations. The (*t*^toc^, *y*^toc^) values are obtained from respective 2SEP^closo^(*n*) ± 2 electron counts and average vertex degrees  of *n*-vertex deltahedra using the TORI approach. The column denoted “Octet rule fulfillment” indicates, cluster-wise (clust.) or the local octet-rule (loc.) fulfillment, respectively.

##### 2.3.4.1 B_4_H_4_ and Si_4_^4–^

The tetrahedral clusters of hypothetical B_4_H_4_ (formally isoelectronic to B_4_Cl_4_) and Si_4_^4–^are special cases of two isostructural clusters, of which the former follows the Wade’s rules (classifying it as a *hypercloso* cluster) and the latter following the classical 8–*N* octet rule.

For the Si_4_^4–^ tetrahedron with 2SEP = 12 ve, the TORI result γ(4) = 0 and *t*^toc^ = 0, *y*^toc^ = 12 ve obtained indicates six classical 2-center 2-electron bonds for the six edges of the tetrahedron,
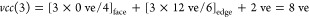
proving local octet-rule fulfillment (note: homogeneous occupations used because of symmetry). This is fully compatible with the Zintl–Klemm pseudoatom approach: 3-bonded Si^1–^, denoted (3b)Si^1–^, is isoelectronic to (3b)P forming 3 local 2-center 2-electron bonds and one lone pair of electrons to fulfill the 8–*N* octet rule. For this purely edge-localized case with 2 exo electrons (one lone pair) and all faces are triangular, the TORI approach is consistent with the Lewis 8–*N* rule as well.

For the B_4_H_4_ cluster with 2SEP = 8 ve, TORI yields γ(4) = 1 and *t*^oct^ = 8 ve, *y*^oct^ = 0 ve, for which local octet-rule fulfillment with three local 3-center 2-electron bonds is obtained:
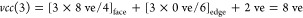


This corresponds to the purely face-localized case of bonding.^16^

##### 2.3.4.2 B_6_H_6_^2–^ and B_12_H_12_^2–^

**Figure 3:**
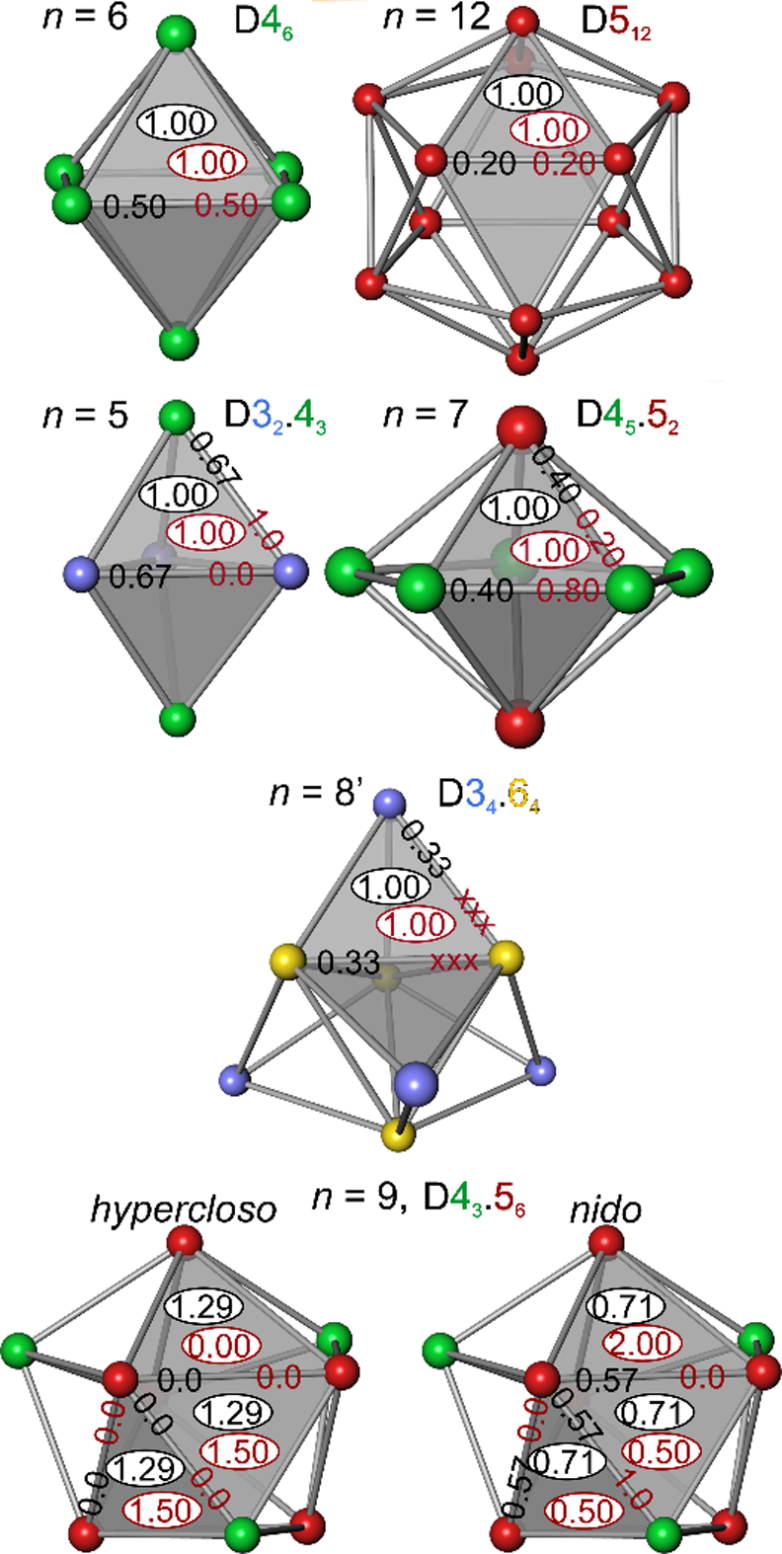
Cluster-wise and local octet-rule fulfillment for *closo* octahedral (*n* = 6), icosahedral (*n* = 12), trigonal bipyramidal (*n* = 5), pentagonal bipyramidal (*n* = 7), omnicapped tetrahedral (*n* = 8′) cluster skeletons, and tricapped trigonal prismatic (*n* = 9) skeletons with *hypercloso* and *nido* SEP counts. The values (in ve) give face-occupation (elliptical frame) and edge-occupation numbers (without frame); black values indicate homogeneous occupations; dark red values indicate local octet-rule fulfilling values.

As the next instructive example, the case of the octahedral skeleton of B_6_H_6_^2–^ (Figure 3, *n* = 6 case) is discussed explicitly. Treatment of B_12_H_12_^2–^ is completely similar, numerical results are different (see Figure 3, *n* = 12). With SEP = 7, the skeletal electron count 2SEP = 14 ve could in principle be completely distributed on all 8 faces, which yields 1.75 ve per face. Since each degree-4 vertex is touched by 4 faces (*vd* = 4) and including the omnipresent exo bond, a local (eq 21) and cluster-wise vertex-configuration count (eq 20) of
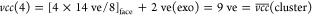
is obtained, which overshoots the local and cluster-wise octet value by 1 ve. The identity of the local and cluster-wise vertex-configuration counts for this highly symmetric case, is explained by the identity of the corresponding local and cluster-averaged vertex degrees .

On the other hand, if 14 ve are distributed on 12 edges, for each degree-4 vertex a value of
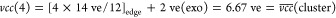
is obtained, which under-fulfills both the local and the cluster-wise octet value. Thus, the suitable mixture of edge and face bonding with mixing coefficient γ(6) = 4/7 ≈ 0.571 (eq 15) yields *t*^toc^ = 8 ve, and *y*^toc^ = 6 ve (eqs 16 and 17), which yields both cluster-wise and local octet-rule fulfillment:
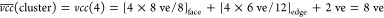
In this highly symmetrical example, where all faces and edges are symmetrically equivalent, the total face (*t*^toc^) and edge (*y*^toc^) occupation numbers have been homogenously distributed among the 8 faces (1 ve per triangular face) and 12 edges (0.5 ve per edge) of the octahedron, respectively. This is indicated in Figure 3 (*n* = 6 case) by the identical values of cluster-wise fulfillment already from homogeneous face and edge occupations (black font) and the specific local octet-rule fulfilling (dark red font) electronic face and edge occupations. For lower symmetries, this is not necessarily the case.

Another important aspect to recognize here is that the description of the local vertex situation already contains the effect of all possible resonances of the 3-center and 2-center 2-electron bond allocations in the *styx* model, which leads to fractional local bond occupations. Thus, each triangular face (3-center bonding) is found to contribute 1.0 ve (i.e., half of an electron pair) to the electronic vertex-configuration count *vcc*(4), and each edge (2-center bonding) contributes 0.5 ve. This is consistent with the general notion of delocalized bonding in these clusters.^21^ Remarkably, this feature does not necessarily impede octet-rule fulfillment. Conversely, it is the specific fractional bonding pattern that causes the fulfillment of the octet rule in these clusters.

##### 2.3.4.3 B_5_H_5_^2–^ and B_7_H_7_^2–^

The aspects of cluster-wise and local octet-rule fulfillments in a specific topological framework are illustrated with two clusters that display 2 different types of vertices and only one type of triangle, e.g. the 5-vertex trigonal bipyramid of B_5_H_5_^2–^ with vertex composition D3_2_.4_3_ (Figure 2). The values *t*^toc^ = 6 ve, *y*^toc^ = 6 ve are obtained. Using homogeneous occupations of all faces (*t*^toc^ /6) and edges (*y*^toc^ /9), respectively, the electronic face-, edge-, and exobond occupation numbers (Figure 3, *n* = 5 case) of the degree-3 vertex, are summed to the following vertex-configuration count:
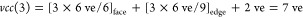


For the degree-4 vertices the following vertex-configuration count is obtained:
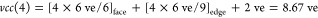
Thus, employing homogeneous occupations none of the vertex types actually seems to fulfill the local octet rule (*vcc*(v_*i*_) = 8 ve) in this framework. But on the cluster-average (eq 25), given by weighting the different vertex-configuration counts obtained by their frequency of occurrence, the value of
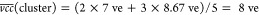
confirms that the octet rule is cluster-wise fulfilled. However, this does not mean that in the delocalized bond picture of the TORI approach the local octet rule has necessarily vanished, and only the cluster-wise one has remained. In fact, the previous calculation did not exploit one degree of freedom in assigning the electronic edge-occupation values. All edges have been given the same value of electronic edge occupation *eocc* = 6 ve /9 despite that there are two different kinds of edges, the six apical–equatorial (ap–eq), and the three equatorial–equatorial (eq–eq) ones. In the present case, the assignment of electronic occupation values for these edges is uniquely determined (and that is why this type of cluster was chosen as an example) by the local octet rule. With the homogeneous assignment of the electronic configuration of the degree-3 vertex shown before, 7 electrons were finally assigned. Since all faces are the same in the cluster, and the degree-3 vertex is connected only to one type of edges (ap–eq), there is only one unique way to define edge occupation *eocc*(ap–eq) such that the degree-3 vertex fulfills the local octet rule, namely *eocc*(ap–eq) = 1 ve (Figure 3, *n* = 5 case). This yields the local octet vertex-configuration count
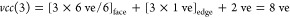


Since all edge occupations in the skeleton follow a sum rule (eq 24) 6*eocc*(ap–eq) + 3*eocc*(eq–eq) = *y*^toc^ = 6 ve, a value of *eocc*(eq–eq) = 0 ve is obtained. The assignment chosen has consequences for the other type of vertices. The degree-4 vertices are connected by two (ap–eq) and two (eq–eq) types of edges. Taking the assigned occupations into account results in a local octet vertex-configuration as well,
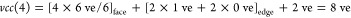


The same strategy applies to the 7-vertex pentagonal bipyramid type of cluster as well. It also exhibits only one type of triangle and two types of edges, and with the apical atom being only connected to one type of edge. The homogeneous assignment of the electronic edge occupation number (Figure 3, *n* = 7 case) leads to an octet under-fulfillment and overshooting for the degree-4 and degree-5 vertices, respectively, and a cluster-wise fulfillment of the octet rule. The adjustment of e*occ*(ap–eq) for the apical vertex to fulfill the octet rule automatically leads to fulfillment for the equatorial vertices as well,
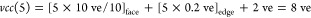

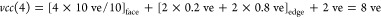
It is worth noting, that for the cases discussed, the edge-occupation numbers derived for fulfillment of the local octet rule in the TORI framework are directly related to the localized 2- and 3-center 2-electron bond allocations for B_5_H_5_^2–^ of Wade.^13^ For example, the value of *eocc*(eq–eq) = 0 ve for this skeleton is indicated in his *styx* (0, 3, 3, 0) type of bond allocation for the *closo* electron count, because there is no eq–eq 2-center 2-electron bond allocated. The *y*^toc^ = 6 electrons have to be assigned solely to the 6 ap–eq edges giving *eocc*(ap–eq) = 1 ve as derived above in the bond-delocalized TORI framework.

The following example illustrates, that the cluster-wise octet-rule fulfillment does not represent a sufficient condition for local octet-rule fulfillment.

##### 2.3.4.4 [Ge_8_]^2–^

In a density-functional study of bare Germanium clusters [Ge_*n*_]^2–^ with *closo* electron count, an 8-vertex cluster with a geometric structure (*T*_*d*_ symmetry, vertex composition D3_4_.6_4_, shown in Figure 3, *n* = 8′ case) different from the boron hydride one (*D*_2*d*_, D4_4_.5_4_, Figure 2) was found to display the minimum total energy being slightly more stable than the prototypical boron hydride type one.^29^ The structure of this cluster skeleton displays a similar situation as the *n* = 5 and 7 clusters discussed above. All 12 deltahedral surface triangles are identical, and there are two different types of edges (of frequencies 12 and 6), where the degree-3 vertices are connected to only one type of edge (frequency 12). With *t*^toc^ = 12 ve, *y*^toc^ = 6 ve (compatible to *styx* code (0, 6, 3, 0) with SEP = 9), the electronic vertex-configuration count using equal edge occupations *occ*(equal) = 6 ve/18 = 0.33 ve (Figure 3, *n* = 8′ case) is computed according to
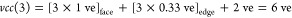


For the degree-6 vertices one obtains
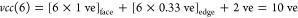
Despite local octet-rule breaking obtained with homogeneous filling the cluster-averaged vertex-configuration count
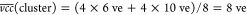
confirms cluster-wise octet-rule fulfillment. If one tries to adjust the electronic edge occupations for the degree-3 vertex to achieve an octet, then a value of *eocc* = 1.0 ve had to be chosen:
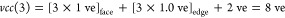
This value is not possible, since the 12 edges of this kind would need 12 ve in total, which contradicts the *y*^toct^ = 6 ve sum rule (eq 24). Thus, local octet-rule fulfillment is not possible in this case. Although the [Ge_8_]^2–^ cluster has the same SEP as B_8_H_8_^2–^ and was therefore classified being isoelectronic to it,^30^ it is not considered as isoelectronic in the TORI approach. This is an important observation, because in the density functional calculations this topology is energetically preferred over the classical boron hydride type skeletal shape (Figure 2), despite its nonconformance with the local octet rule. It can be interpreted that the cluster-wise octet-rule fulfillment is actually more important than the local one.

In general, the advantages of the cluster-wise octet-rule fulfillment over the local one is the possible (if symmetry permits) appearance of additional local electronic and geometrical degrees of freedom, which need only be compensated cluster-wise. If this reasoning is generally valid for other clusters, then TORI would be able to identify the essential conceptual difference between the 8–*N* type polyanions and the deltahedral Wade clusters: the localized bond allocations leading to local octet-rule fulfillment in the first case, and the dominating cluster-wise octet-rule fulfillment in the second case.

##### 2.3.4.5 Deltahedral Skeletons for Different Non-closo 2SEP Counts: B_9_Cl_9_ and [Bi_9_]^5+^

The similar deltahedral cluster skeletons of both clusters with different, *hypercloso* (B_9_Cl_9_)^31^ and *nido* ([Bi_9_]^5+^)^27^ electron counts, respectively, are famous examples. They most clearly reveal the main difference between the 8–*N* type polyanions with localized 2-center 2-electron bonding, where the dominating local octet rule implies different partial structures for different electron counts, and the Wade-type of clusters, where the delocalized type of bonding leads to octet-rule fulfillment of the same partial structure (cluster skeleton topology) with different electron counts.

Since both clusters display deltahedral skeletons, the TORI approach is applicable in the present form. Both clusters display the same 9-vertex skeleton with vertex composition of D4_3_.5_6_ and *n*_F_ = 12 + 2 and *n*_E_ = 12 + 6 + 3 (like B_9_H_9_^2–^, Figure 2).

For B_9_Cl_9_ with 2SEP = 18 ve, the values *t*^toc^ = 18 ve, and *y*^toc^ = 0 are obtained (Table 1). This yields a homogeneous face occupation of *focc* = 18 ve /14 = 1.29 ve per face and zero edge occupations throughout. With this choice, the degree-4 and 5 vertices exhibit vertex- configuration counts of
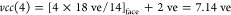

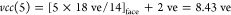
respectively. The cluster-averaged vertex-configuration count proves cluster-wise octet-rule fulfillment:
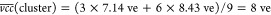
The local octet-rule fulfilling values for the 2 different kinds of 14 triangles (12 + 2) are easily obtained using the fact, that the degree-4 vertices only have one kind of triangles (12 of type 1 in total), whose triangle occupations can then be adjusted to *focc*(type 1) = 1.5 ve in order to locally fulfill the vertex-4 atoms’ octet rule
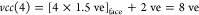
This leaves 18 ve – 12 × 1.5 ve = 0 ve (sum rule eq 23) for occupation *focc*(type 2) of the two remaining triangular faces of type 2, which is fully consistent with Wade’s bond allocation.^16^ In sum, a vertex-configuration count for degree-5 vertices of
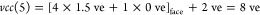
is obtained confirming local octet-rule fulfillment for this type of cluster.

For the deltahedral cluster [Bi_9_]^5+^ with *nido* skeletal electron count, values *t*^toc^ = 10 ve, and *y*^toc^ = 12 ve are obtained (Table 1). Because of eq 20, also this cluster displays cluster-wise octet-rule fulfillment:
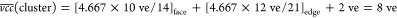


Assessing the local octet-rule fulfillment is more difficult than in the *hypercloso* and the other cases discussed above, because the two types of vertices in this deltahedron display a more variable coordination by 2 different faces and 3 different edges. In this case, the pertinent face and edge occupations are derived from the allocated bond distributions for this type of cluster with *nido* electron count.^16^ This way, fractional face and edge occupations are obtained, which yield local octet-rule fulfillment for both vertex types. For the degree-4 vertices the following sum of face and edge occupations is obtained:
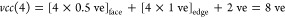


This leaves for the other type of face 10 ve – 12 × 0.5 ve = 4 ve (i.e., 2 ve per such a face), and the other two types of edges display no occupations: 12 ve – 12 × 1 ve = 0 ve. Thus,
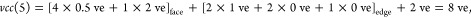
which confirms the local octet-rule fulfillment in the TORI framework also for the *closo*-shaped *nido*-electronic [Bi_9_]^5*+*^ cluster.

Thus, both isostructural clusters *hypercloso* (B_9_Cl_9_) and *nido* ([Bi_9_]^5+^) display cluster-wise and local octet-rule fulfillment despite their different 2SEP numbers. This behavior is not known from those Zintl-phases with partial structures following the local 8–*N* type of octet rule.

## 3 Conclusions

The Topological Octet Rule Implementation (TORI) approach opens a new way to expand the basic Lipscomb’s model construction of localized 2c-2e and 3c-2e bonds by dropping the 2-electron component, which is finally lost even in the *styx* framework after the necessary resonance averaging. In contrast, the TORI approach argues within a delocalized 2- and 3-center bonding framework in deltahedral clusters. Nevertheless, the sums of fractional 2- and 3-center bond occupations *y*^toc^ and *t*^toc^, respectively, obtained from TORI are consistent with the *styx* values of localized 2- and 3-center 2-electron bonds. Thus, despite starting from complementary initial assumptions, a consistent overall picture is obtained, which contains facets of both approaches.

One important difference to the *styx* approach is that two different types of octet-rule fulfilment have been observed in the TORI framework, the cluster-wise and the more stringent local one. The deltahedral Wade-type boron hydride clusters with 2SEP^*closo*^ counts are found to obey both, the cluster-wise and the local one for each vertex type. However, the cluster-wise octet-rule fulfillment is not a sufficient condition for the local octet-rule fulfillment. Cluster-wise octet-rule fulfillment for deltahedral clusters of *hypercloso*, *closo*, and *nido* 2SEP counts is guaranteed within the TORI approach.

For deltahedral clusters, the cluster-wise octet-rule fulfillment may be even more important than the local one. The advantage of the cluster-wise octet-rule fulfillment over the local one is the symmetry-dependent appearance of additional local electronic and geometrical degrees of freedom with respect to octet-rule fulfillment, which need only be compensated cluster-wise. In the highly symmetrical boron hydride clusters *n* = 6, 12 local and cluster-wise octet-rule fulfillment work in parallel, and these clusters are found to be the most stable ones with respect to their size *n* of the series *n* = 5 to 12.^34^ For the remaining *closo* clusters a preference for the most homogeneous skeletal shapes with most similar vertex degrees is well-known. This may be interpreted as the topological preference of small differences between local and cluster-wise octet-rule fulfillment.

In the TORI approach, the main difference between 8–*N* type of Zintl polyanions and the Wade’s type of clusters is found in the more flexible way of octet-rule fulfillment in the latter type of compounds. It may lead to octet-rule fulfillment, both, local and cluster-wise, of the same deltahedron (partial structure) with different skeletal electron counts. In contrast, the 8–*N* rule for Zintl–Klemm types of polyanions allows only one unique electron count for a specific partial structure.

By trend Wade clusters in the *hypercloso*, *closo*, *nido*, *arachno*, ... sequence gradually turn to molecules, where the local octet-rule fulfillment finally becomes dominant as documented by the success of the Zintl–Klemm pseudoatom approach.

The TORI approach works with delocalized fractional bonds and is consistent with the concept of PSEPT. It just adds an additional facet. Moreover, because of TORI’s relation to the styx approach, it may be considered to build a bridge between the styx approach and PSEPT.

## Notes

The authors got notice of a related paper on the same subject.^35^ In the region of topical overlap, both papers agree with each other.^35^
